# Temporary Paralysis of Peristalsis Following Injuries by Trolley

**Published:** 1895-07

**Authors:** W. Horace Hoskins


### REPORTS OF CASES.

### Temporary Paralysis of Peristalsis Following Injuries by Trolley.

BY W. HORACE HOSKINS.

The subject, a gray gelding, about thirteen years old, had been in the possession of present owner about eight years, and never lost a day from sickness in that period.

On the evening of June 4th, while being ridden from the blacksmith shop, was struck by a trolley-car on the right hip and croup, knocked down and pushed along by the car some thirty feet. On regaining its feet did not seem to be much hurt; was walked to its stable and subsequently fed. Feed, as well as hay, remained uneaten in the morning, when the horse was found in slight pain, and evidences presented themselves indicating that it had been uncomfortable during the night.

On the morning of the 5th the horse was brought to my sanitarium, and an examination revealed the complete absence of sound indicating peristalsis, with some loss of power in the hind quarters, and recurring spasmodic attacks of pain, horse lying down, pawing, and looking around at the side. An anodyne drench was given, and temporary relief followed shortly after, but the pain recurred again and again every two or three hours for some three days. After the third dose was given the anodyne mixture was administered in pint draughts of linseed oil, until some two quarts of oil had been given.

After the first few hours in the hospital, during which one or two small droppings of manure occurred, accompanied with much difficulty in voiding and arching the back, no fecal matter was voided for some seventy-two hours. After waiting for the action of the oil an eight-drachm ball of aloes was administered, which, in twenty-four hours, gave rise to much uneasiness and distress, followed by ineffectual efforts to unload the lower bowel. In addition to above treatment clysters of glycerin were repeatedly tried ineffectually. Some thirty or more hours after administration of aloes ball, indications of inability to unload the rectum were evident, and an examination revealed the existence there of a large mass of dry fecal matter distending the rectum to an unusual extent. For some forty-eight hours all fecal matter reaching the rectum had to be removed by hand. When received at the sanitarium, on the 5th, the horse weighed 1060 pounds, on the 10th weighed 1035 pounds, was discharged on the 15th, weighing 982 pounds, with instructions to give light work for a week.

